# Nutritional anthropology in the world

**DOI:** 10.1186/s40101-023-00345-0

**Published:** 2024-03-08

**Authors:** Stanley Ulijaszek

**Affiliations:** Unit for BioCultural Variation and Obesity, School of Anthropology and Museum Ethnography, University of Oxford, Oxford, England

**Keywords:** Nutrition, Food, Anthropology, Diet, Evolution, Ecology, Biocultural

## Abstract

Nutritional anthropology is the study of human subsistence, diet and nutrition in comparative social and evolutionary perspective. Many factors influence the nutritional health and well-being of populations, including evolutionary, ecological, social, cultural and historical ones. Most usually, biocultural approaches are used in nutritional anthropology, incorporating methods and theory from social science as well as nutritional and evolutionary science. This review describes approaches used in the nutritional anthropology of past and present-day societies. Issues of concern for nutritional anthropology in the world now include: understanding how undernutrition and food insecurity are produced at local, regional and international levels; how food systems are constructed using social, biological and biocultural perspectives; and obesity from a biocultural viewpoint. By critiquing framings of present-day diet in an evolutionary context, nutritional anthropology asks ‘what should be eaten?’, rather than ‘what can be eaten?’, and ‘how cheaply can people be fed?’.

## Background

Nutritional anthropology is concerned with understanding human subsistence, diet and nutrition from both comparative social and evolutionary perspectives. Diet and subsistence have been of interest to anthropologists in reporting on the life of different societies since the late nineteenth century, while studies of nutrition in social context began in the 1930s [1], with studies of hunger, land use and diet in Northern Rhodesia by Audrey Richards [2, 3]. Studies of diet, subsistence and nutritional health became increasingly ecological in subsequent decades [4], especially with the emergence of evolutionary ecology as a discipline [5] which stimulated biological anthropological approaches to human evolution foregrounding nutrition, foraging and subsistence [1]. These and other social and human evolutionary food and nutrition-related fields came together as nutritional anthropology in the 1980s [6]. Using integrated biocultural approaches, nutritional anthropology also took within its remit ecological investigations of undernutrition [7] and obesity [8, 9], as well as study of localised food systems [10], food security [11], infant feeding and adaptation [12], the political ecologies of dietary change and nutritional health, in the contexts of globalisation, migration, inequality [11] and colonialism [13]. Nutritional anthropology has thus developed a varied set of approaches to the entangled and changing relationships of humans, and the societies they live in, with food and nutrition.

## Patterns of dietary change

In nutritional terms, humans are the sum total of their evolutionary history and more recent epigenetic and social past, as well as their present-day social, cultural and biological life histories. Human societies have undergone many transformations, most of which have included transitions in diet and nutritional health, and nutritional anthropologists have documented such changes in many societies [7]. For most of their evolutionary past, humans have been foragers, subsisting on plants, fungi, Protista, bacteria and animals gathered and hunted in the wild, ancestral hominins having been able to subsist on a diversity of food resources from a wide range of trophic levels. The emergence of cooking in the Paleolithic Period transformed dietary quality [14], making foods more digestible and palatable while reducing their potential toxicity. Fundamental to the survival and success of prehistoric humans were their abilities in finding and competing for high-quality foods in most environments (meat, honey, eggs and berries, for example) as well as their developing food processing techniques which allowed food to be made more digestible [7].

The transition to agriculture in the Neolithic period saw widespread adoption of crop planting and animal husbandry in several centres across the world, more or less concurrently [15]. The intensification of food production at this time was associated with reduced dietary diversity, increased work loads in subsistence, specialisation of economic production, and inequality [7]. Seasonality, whether of rainfall or temperature, or both, which had earlier guided subsistence patterns for hunter-gatherers, came to systematise work patterns for agriculturalists according to the types of plants and animals they domesticated [1]. This set the pattern for agricultural production that has persisted to the present day, even with many technological changes that have come since then.

Permanent, year-round settlements were a feature of the new agricultural societies, with people living together in close proximity allowing existing pathogens to intensify their infection of humans, and for animal-borne diseases to cross the species barrier into humans, creating new ecological problems which related to nutrition, in the form of undernutrition-infection interactions [16]. Population settlement allowed humans to live in a more material world than ever before, allowing the ascription of value to goods beyond their immediate functional value, also mobilising them for marking social distinction. Food, the most primary of symbolic goods, is likely to have been used symbolically as well as materially, especially in relation to meat [17]. The social use of foods has continued to the present day, is found in all societies, and is often related to nutritional health [7]. For nutritional anthropologists, understanding the deep social embeddedness of food as a symbolic good enriches interpretations of how nutritional health is produced in society.

## Past diet informing the present

Modern food systems carry vestiges of the past, the dominant products of agriculture now eaten in the world being mostly a small number of cereal types and animal species, selected by the practices of traditional farming and animal husbandry, and more recently by scientifically-informed production. Humans have used their technological abilities to harvest, process and consume a very wide range of food-yielding plants and animals in a globally extensive range of environments [18]. Inter- and intra-individual dietary flexibility has deep evolutionary roots, and has permitted survival and success in many contrasting circumstances. Understanding how foraging strategies have shaped survivorship, health and well-being as well as reproductive success among past hominin and human populations has helped understandings of human eating practices and feeding behaviours now. Since human form and function change slowly and must be able to respond to rapidly altering dietary circumstances, one approach in nutritional anthropology places emphasis on evolved predispositions that are either adaptive or become maladaptive with changing contexts. Energy has usually been the major limiting factor in foraging, and the study of energy-linked reproductive fitness has helped identify how the drive for macronutrient consumption has shaped (and continues to shape) overall diet quality [19]. The preference for energy-dense foods (often dense in protein too, as with meat and fish) for assuaging hunger would have reinforced the importance of energy-dense foods in the past and contributed to their attractiveness in the present day.

Understanding the evolution of the human diet informs debates concerning what humans should eat now. The idea of the ‘Stone Age diet’ (also known as the Paleo Diet), was first put forward by Eaton and Konner [20], who argued that what people ate in the Paleolithic Period, in what has been considered to be the environment of evolutionary adaptedness [21], was more closely attuned to human dietary and nutritional needs than at any period since. By collating and quantifying nutrient intakes of contemporary hunter-gatherer groups using the Human Relations Area Files [22], and showing how similar averaged estimates of intakes of such groups are to the USA-recommended nutritional guidelines of the 1980s, Eaton and Konner [20] proposed that dietary recommendations for good health should be based on the Paleolithic ideal—thus, the Stone Age diet. The ideas underpinning the Stone Age diet have gained popular traction since then because they offer the prospect of being able to consume a natural diet that humans are viewed to be best adapted to, and which is therefore assumed to be the most healthy. Nutritional anthropology has critiqued suppositions that underpin the Stone Age diet, using evolutionary and ethnographic frameworks. As an idea, the Stone Age diet has stimulated critiques of present-day nutritional norms and contemporary industrialised diets [23], food guide pyramids for the US prior to the 2000s having been deconstructed as representing political, rather than health, interests [24]. Diverse diets in the Paleolithic Period are likely to have allowed the intake of quantities of most micronutrients at levels several-fold greater than the US recommended daily allowances of the 1980s and of Reference Daily Intakes of the present time. According to the proponents of the Stone Age diet, these higher intakes may be much more in tune with good health than the minimum requirements recommended for most nutrient intakes in most guidelines for nutritional health. Against this, the setting of minimum levels of nutrient intakes for populations has helped many countries attain good or acceptable food security, by underpinning food production goals and creating the conditions for the industrialisation of food based on scientific principles.

There have been various reformulations and assessments of the Stone Age, or Paleo, diet [25, 26], and it has continued to be a focus for evolutionarily-based critique of present-day industrial foods [7]. Developments in paleoanthropology, population genetics and epidemiology have increased certainty about evolutionary processes related to diet, the time-frames in which they took place, and how they inform our knowledge of contemporary human diet [27, 28], and anthropology of food and nutrition continues to feed the debate about what humans should eat and how [29].

Since the Paleolithic Period, cooking would have continued to help establish regional and local traditions, preferences and eating behaviors with population expansions across the world. Some early cultivated crops and domesticates, such as squash and maize in Mesoamerica, chickpeas in the Middle East, rice and pork in Southeast Asia and taro and banana in New Guinea, remain distinctive elements of the cuisines of these regions to the present day. Understanding how foods have been taken up and used by different groups since the origins of agriculture and how diets have become increasingly cosmopolitan in the past few hundred years are key issues for understanding diets, food security and patterns of nutritional health now. Most recently the consumption and social use of novel foods has also become an area of investigation for nutritional anthropology [30].

## Food and nutritional health

The anthropological understanding of food in its social and ecological contexts offers an alternative perspective to the functionalist one of food as a vehicle for the delivery of nutrition. It shows how food production, supply and consumption are all highly political [24], and how poverty and inequality manifest themselves in undernutrition [31] and overnutrition [32], respectively. Undernutrition and obesity often sit side by side within the same communities, and/or households, in what has been described as the dual burden of under- and over-nutrition [33].

Phenotypic plasticity, manifest in phenomena such as low birth weight, growth faltering and subsequent catch-up growth [34], enables the exploitation of changing and changeable environments for food while minimising mortality due to hunger. While fundamental to human survivorship and success in the past, phenotypic plasticity has become maladaptive in many parts of the world now, where epidemiological and nutrition transitions have been taking place, where plastic responses to poor early life environments leave people more predisposed to chronic disease later in life [35]. The ‘developmental origins of health and disease’ concept hypothesises that chronic diseases that develop later in life originate in the fetus by environmental programming [36]. Epigenetic regulation during fetal programming [37] prepares an individual for the environment they expect to enter at birth and beyond, but when there are changes in nutritional circumstances, chronic disease in adult life is often the result. Humans can respond to changing dietary and nutritional circumstances through genetics (via natural selection), phenotype, via developmental plasticity, and epigenetics (by a balance of both). Removal of dietary energy stress and uncertainty, as has taken place in many societies, turns these adaptive processes toward pathology. An area of promise for nutritional anthropology lies in understanding how social processes can shape genetic expression [38] in the construction of nutritional health and disease [36].

Undernutrition and obesity are both manifestations of extreme energy imbalance at ecological, societal and individual levels. In past populations and among societies practicing traditional subsistence now, fluctuations in energy balance have been, and continue to be the norm, especially with seasonal swings in food availability [1]. Non-seasonal, long-term food shortages were also experienced in the past, reflecting ecological stress due to a range of factors including climate change and over-foraging of resource areas. Long-term positive energy balance is not usually found in nature, but nor is obesity. Modern human populations, with assured food supplies and controlled reproduction, are able to express an uncommon trait, that of prolonged and extreme body fatness, often to the point of pathology [39].

Political-economic factors frame nutritional health at local and individual levels by determining what foods are available to buy, and at what prices, economic inequality being a major determinant of what can and can’t be consumed. The industrialised food system has penetrated most parts of the world now and promotes consumption often in ways that promote poor nutritional health, as the foods that the industry produces in greatest volume are energy-dense and usually high in refined carbohydrates including sugar, and fat [40]. In present-day industrialised societies, energy intake is regulated less by availability and more by cognitive restraint by individuals, there being very weak physiological homeostatic mechanisms to prevent weight gain. Human eating decisions concerning what to eat, when to eat and how to eat, are powerfully influenced by social contexts. By eating meals together, humans embed feeding in social structures based on kinship and household temporal organisation. Meals are central to social structure and group identity, and an anthropological understanding of obesity includes the study of social patterning of food consumption [41], as well as an appreciation of how body fatness is valued in society [42], both of which are concerns for nutritional anthropology.

## Conclusions

Food and nutrition shape many aspects of humanity, including identity, reproduction, sociality, as well as health and well-being. Nutritional anthropology critiques framings of present-day diet in an evolutionary context and asks ‘what should we be eaten?’ rather than ‘what can be eaten?’ and ‘how cheaply can human populations be fed?’ Informed by evolutionary and biocultural approaches to human diet, answers to these questions are important, at this time of global dietary change and unstable food security. Undernutrition and obesity persist in the world and are increasing in many places, making the understanding of food and nutrition in evolutionary, ecological, and social contexts as important now as when nutritional anthropology first emerged as a sub-discipline.

## Data Availability

Literature and library sources all obtained by SU.

## Abbreviation

USA: United States of America
